# Drug discovery of anticancer drugs targeting methylenetetrahydrofolate dehydrogenase 2

**DOI:** 10.1016/j.heliyon.2018.e01021

**Published:** 2018-12-17

**Authors:** Ayumu Asai, Jun Koseki, Masamitsu Konno, Tatsunori Nishimura, Noriko Gotoh, Taroh Satoh, Yuichiro Doki, Masaki Mori, Hideshi Ishii

**Affiliations:** a Department of Medical Data Science, Graduate School of Medicine, Osaka University, Suita 565-0871, Japan; b Department of Frontier Science for Cancer and Chemotherapy, Graduate School of Medicine, Osaka University, Suita 565-0871, Japan; c Department of Gastroenterological Surgery Graduate School of Medicine, Osaka University, Suita 565-0871, Japan; d Division of Cancer Cell Biology, Cancer Research Institute, Kanazawa University, Kanazawa 920-1192, Japan; e Department of Surgery and Science, Graduate School of Medical Sciences, Kyushu University, Fukuoka 812-8582, Japan

**Keywords:** Cancer research, Pharmaceutical science, Molecular biology

## Abstract

Many anticancer drugs have serious adverse effects; therefore, it is necessary to target features specific to cancer cells to minimize the effects on healthy cells. Methylenetetrahydrofolate dehydrogenase 2 (MTHFD2) was reported to be specifically enhanced in cancer. We confirmed the validity of MTHFD2 as a drug discovery target using clinical data. In addition, we performed *in silico* screening to design an anticancer drug specifically targeting MTHFD2. Analysis of the clinical data indicated that MTHFD2 was enhanced in most cancers compared with normal tissues, and affected the prognosis in cancer patients. Candidate compounds for MTHFD2 inhibitors were identified using *in silico* drug discovery techniques, and the important interactions for MTHFD2 binding were determined. In addition, these candidate compounds decreased levels of MTHFD2 metabolites in cancer cells. The findings of the present study may help to develop anticancer drugs targeting MTHFD2, with a view to minimizing the adverse effects of anticancer drugs.

## 1 Introduction

Various anticancer drugs have been designed for cancer therapy. Anticancer drugs directly targeting DNA replication and cell division, such as alkylating agents, topoisomerase inhibitors, and antimicrotubule agents, have serious effects on cancer cells as well as normal cells [1, 2, 3]. Consequently, many anticancer drugs cause serious adverse side effects. Targeting features that are specific to cancer cells can minimize the effects on normal cells. For example, antibody drugs have attracted attention, since they have a high specificity to cancer cells. However, these drugs also present problems such as not being able to access intracellular targets [4]. In addition, the production of antibody drugs requires the use of very large and sophisticated cell cultures, making them expensive [5]. Relatively inexpensive low molecular weight anticancer drugs specific to cancer cells are required for multidrug combination chemotherapy.

**Fig. 1:**
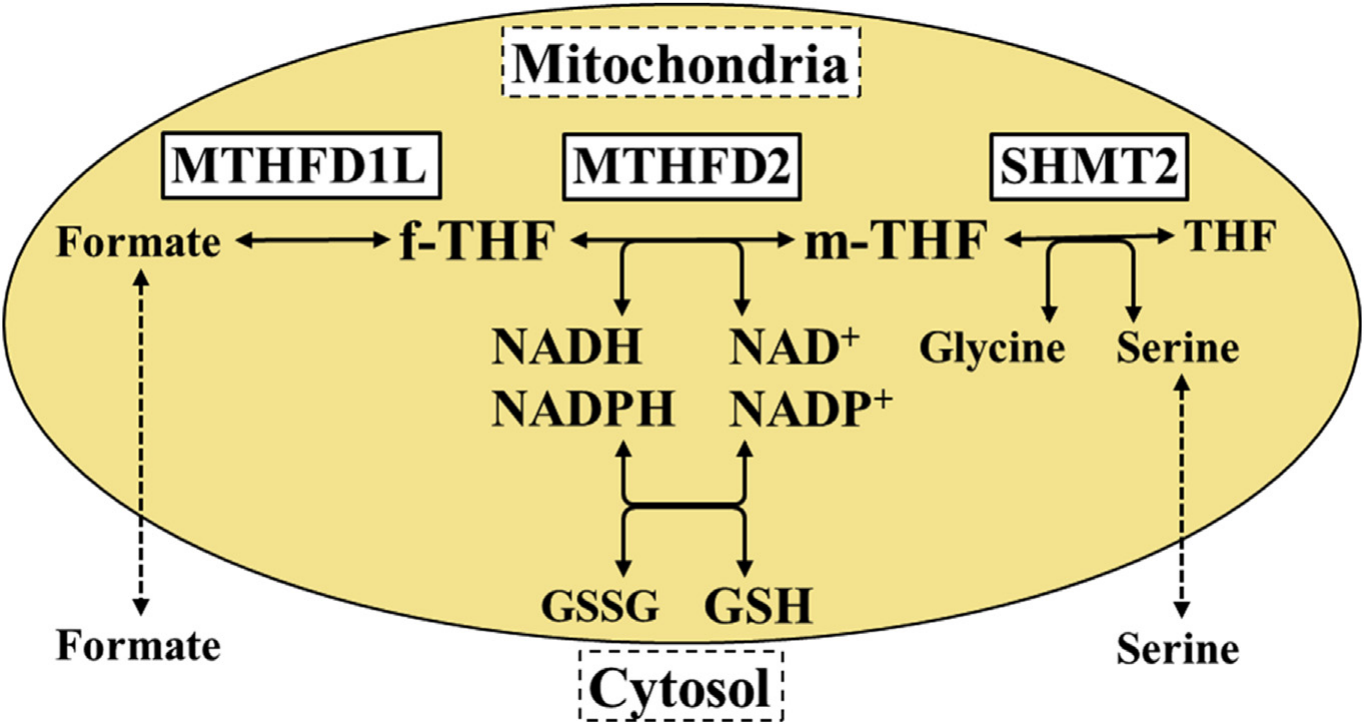
Mitochondrial one-carbon metabolism. Mitochondrial one-carbon metabolism comprises reactions involving enzymes such as MTHFD2 and SHMT2. MTHFD2 catalyzes the reaction between m-THF and f-THF, and is the main enzyme involved in redox.

Cancer cells enhance their proliferation and malignancy by adapting their metabolism [6]. For example, glycolysis, the pentose phosphate pathway, and glutamine metabolism are known to be adapted in cancer cells [7, 8, 9]. However, these metabolic pathways are crucial for normal cells; therefore, cancer therapy targeting these pathways has not yet been established. The one-carbon (C1) metabolism pathway in mitochondria recently attracted attention as a promising target for cancer therapy. C1 metabolism is a pathway composed of a folate cycle and a methionine cycle. This pathway plays roles in DNA synthesis via the production of nucleic acid components [10], redox via the production of nucleic glutathione [11], and methylation via the supply of a carbon source [12]. C1 metabolism occurs in the cytosol and mitochondria. In the cytosol, C1 metabolism was originally used as a drug discovery target, including for thymidylate synthase (TYMS; a target of 5-fluorouracil) and dihydrofolate reductase (DHFR; a target of methotrexate). However, in recent years, it was reported that C1 metabolism in the mitochondria was markedly enhanced in a comprehensive gene analysis of cancer cell lines and tumor tissues of cancer patients, such as serine hydroxymethyltransferase 2 (SHMT2) and methylenetetrahydrofolate dehydrogenase 2 (MTHFD2) [13, 14]. Hence, SHMT2 inhibitors were designed as anticancer drugs [15]. MTHFD2 has potential for cancer therapy as a promising drug discovery target compared with SHMT2 [13, 14, 16]. It is a bifunctional enzyme catalyzing the reaction 5,10-methylene tetrahydrofolate (m-THF) + nicotinamide adenine dinucleotide (phosphate) (NAD(P)) ⇌ 10-formyl tetrahydrofolate (f-THF) + NAD(P)H [17] (Fig. 1). The importance of MTHFD2 has mainly been reported in breast cancer [18, 19]. It was reported that MTHFD2 is involved in proliferation [19, 20], metastasis [18], stemness, and chemoresistance [18] in cancer cells, and suppression of MTHFD2 reduces the malignancy [18, 20]. Therefore, MTHFD2 inhibitors represent a promising candidate for cancer therapy. Importantly, MTHFD2 is expressed in the developing embryo, but is absent in most adult normal tissues [14, 20]. MTHFD2 inhibitors may have fewer detrimental effects on normal healthy cells, which is important to prevent the serious adverse side effects of many anticancer drugs. Although the crystal structure of MTHFD2 was reported [21], no MTHFD2 inhibitors have yet been reported.

Thus, we utilized *in silico* drug discovery techniques to report MTHFD2 inhibitors promptly. A quantitative Structure-Activity (QSAR), ligand based drug discovery (LBDD), and structure based drug discovery (SBDD) are known one of the *in silico* drug discovery method. In particular, LBDD and SBDD methods are widely used. LBDD is a technique for drug design based on some known active compounds for target protein. So it does not require a target protein structures. On the other hand, SBDD is a drug design method based on the enzyme pocket structure of target protein. That's why we can design the candidate compounds with high inhibitory activity and high specific selectivity compared with LBDD in general. No inhibitory compound has been reported for MTHFD2. Thus, for the drug discovery targeting MTHFD2, SBDD is more suitable and we decided to perform it.

In the present study, we used two strategies to develop an anticancer drug targeting MTHFD2. First, we examined MTHFD2 expression in patients with different cancers and analyzed the association between the cancer prognosis and expression of MTHFD2 to validate the possibility of MTHFD2 as a drug discovery target. Second, we identified candidate compounds binding to MTHFD2 and determined the important interactions involved in binding to MTHFD2 using *in silico* drug discovery techniques.

## 2 Materials and methods

### 2.1 Analysis of gene expression and prognosis in patients

We measured MTHFD2 expression in patients with different cancers and compared expression between primary tumors and normal tissue using data from The Cancer Genome Atlas (TCGA) [22]. A box plot diagram was generated using the boxplot package in R (version 3.0.2). Prior to the significance test, the distribution of data was confirmed using an F-test. Statistically significant differences were determined by Student's *t* test (if data were homoscedastic) or Welch's *t* test (if data were heteroscedastic).

Patient data were collected for colorectal cancer using the GSE17536 database [23] (n = 177) and lung adenocarcinoma using the GSE31210 database [24] (n = 226) from the Gene Expression Omnibus at the National Center for Biotechnology Information, and used to analyze the effect of MTHFD2 expression on the overall survival of patients. Kaplan–Meier curves at 5-year follow-up were generated using the survival package in R. Statistically significant differences were determined using a logrank test.

### 2.2 Molecular dynamics simulation

We performed molecular dynamics (MD) simulation in the water phase with the AMBER99 and TIP3P force field for protein and water, respectively, using the AMBER12 program package, to analyze the thermodynamic behavior of MTHFD2. The crystal structure of human MTHFD2 was registered in Protein Data Bank (PDB). The initial coordinate in the MD simulation was identified from the crystal structure of MTHFD2 (PDB ID: 5TC4 [21]), and hydrogen atoms were added. Energy minimization was carried out before the elevated temperature process. We then performed NVT–MD simulation (elevated temperature process and thermodynamically conformational sampling) at around 37.0 °C (310 K) using the periodic boundary condition. The temperature constant was maintained with Langevin dynamics in the NVT-MD simulations.

### 2.3 *In silico* drug discovery

**Fig. 2:**
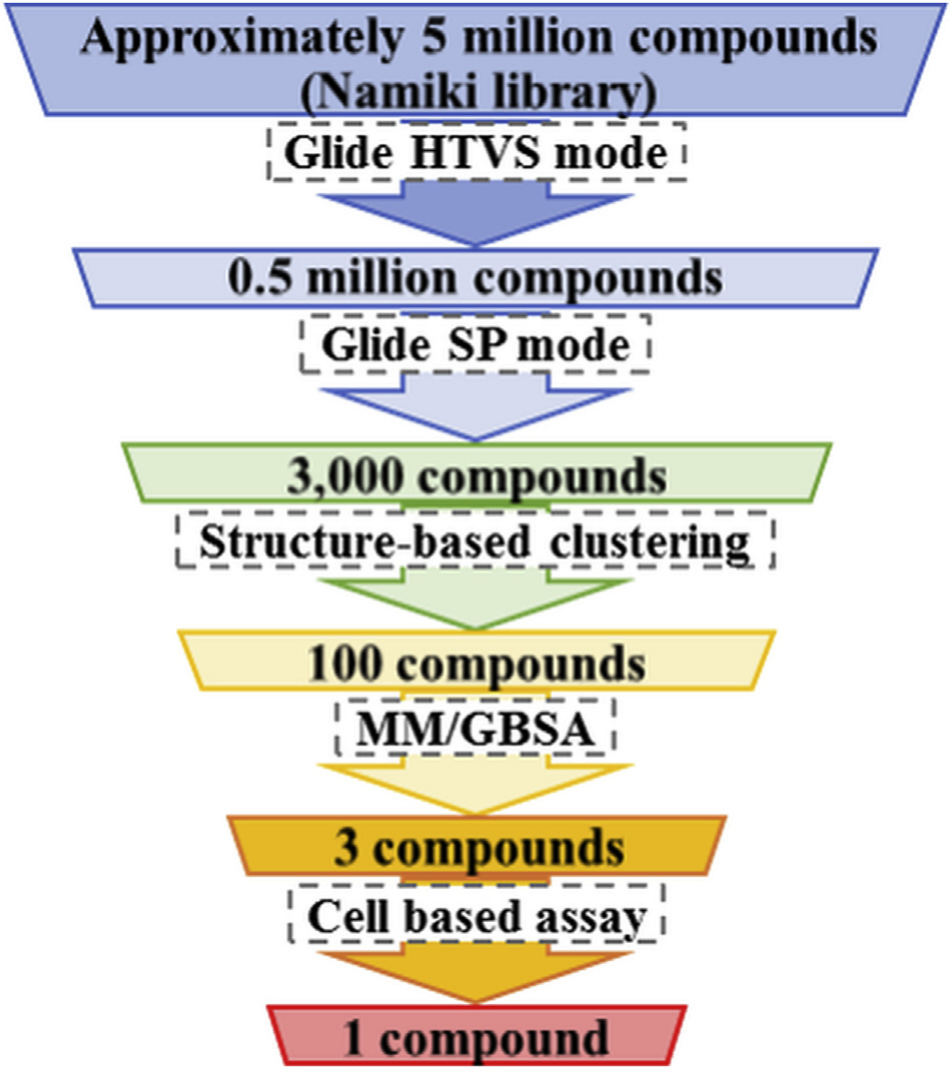
*In silico* screening flow. An *in silico* screening flow was performed to detect candidate compounds targeting MTHFD2 among approximately 5 million compounds using the Schrödinger Suite. Glide high-throughput virtual screening (HTVS; speed emphasis) and standard precision (SP; standard) mode shows the general docking simulation. MM/GBSA shows the docking simulation considering the solvent effect.

Docking simulations were performed for structure-based screening using chemical compounds (approximately 5 million compounds) provided by Namiki Shoji Co. Ltd (Tokyo, Japan). *In silico* screening was performed using Schrödinger Suite 2014 (Schrödinger, LLC, New York, NY, USA). At the first screening, the most common, approximately 0.5 million, compounds were detected using the high-throughput virtual screening (HTVS; speed emphasis) mode of the Glide docking program (Fig. 2). At the second screening, the most common, approximately 3000, compounds were detected using the standard precision (standard) mode of the Glide docking software. Screening was performed using the crystal structure of MTHFD2 (PDB ID: 5TC4 [21]) in the first and second screenings. 100 compounds were detected via clustering to detect the representative structures of candidate compounds identified in the second screening. In parallel, for the tetrahydrofolate (THF) pocket, 26 target conformations were selected from the MD trajectory to investigate thermal variability of protein structure of MTHFD2. Similarly, for the NAD pocket, 9 conformations were selected. Before the molecular mechanics–generalized Born surface area (MM/GBSA), we performed docking simulation for each conformation of MTHFD2 with the 100 compounds detected by the clustering. These candidate compounds were ranked using MM/GBSA with the highest affinity conformation in each compound to investigate the solvent effect at docking.

We performed *in silico* docking simulation with MIT and MIN for MTHFD1 and SHMT2 to confirm specificity of MIT and MIN for MTHFD2. *In silico* docking simulation, the crystal structures of MTHFD1 (PDB ID: 1DIB []) and SHMT2 (PDB ID: 3OU5 []) were used. MIT and MIN were compared with each metabolite (MTHFD1: m-THF, f-THF, NAD and NADP; SHMT2: serine and glycine) and inhibitor (MTHFD1: LY345899; SHMT2: SHIN1 []) for each enzyme using the MM/GBSA.

### 2.4 Cell culture

The DLD-1 human colon cancer cell line was obtained from the American Type Culture Collection (Manassas, VA, USA). Cells were cultured in Dulbecco's modified Eagle's medium (Nakarai Tesque, Kyoto, Japan) supplemented with 10% fetal bovine serum (Thermo Fisher Scientific, MA, USA), at 37 °C in a humidified atmosphere with 5% CO_2_.

### 2.5 Cell-based assay

Confluent DLD-1 cells grown on 6-cm dishes were treated with each candidate compound detected by *in silico* screening at concentrations of 10 or 100 μM. One or four hours after treatment, the cells were detached using TrypLE Express (Thermo Fisher Scientific, MA, USA) and centrifuged to form a cell pellet. The cell pellets were resuspended in 100 μL of extraction buffer (10 g/L ammonium formate, 1 g/L ascorbic acid in water; Wako Pure Chemical Industries, Ltd., Tokyo, Japan). The supernatants were collected by centrifugation at 12,000 × *g* at 4 °C for 15 min (Kubota Co., Tokyo, Japan), then filtered using a 0.45-μm pore sized glass microfiber membrane (Kanto Chemical Co., Inc., Tokyo, Japan).

Next, 10 μL of filtered aliquots were applied to a column (ZORBAX ODS, 4.6 mm × 150 mm, 5.0 μL, Agilent Technologies, Palo Alto, CA, USA) connected with a liquid chromatography system (Agilent 1200 Series), equipped with a triple quadrupole mass spectrometer (Agilent 6410, Agilent Technologies) and an electrospray source at the following settings: column temperature 35 °C, mobile phase A: 0.5% acetic acid, B: 80%:20% methanol:acetonitrile, gradient at 90%A:10%B to 5%A:95%B for 15 min, flow rate at 0.4 mL/min, positive ESI mode at 1850 V, ionization source 30 psig, drying gas temperature 350 °C, and flow rate of 10 L/min. The reagents for the mobile phase were purchased from Wako (acetic acid, methanol, and acetonitrile). The multiple reaction monitoring mode parameters were as follows: 458.3–311.1 *m*/*z* for 5,10-methylenetetrahydrofolate and 474.2–456.3 *m*/*z* for 10-formyltetrahydrofolate. Each detected peak was compared as an amount of the metabolite. Prior to the significance test, the distribution of data was confirmed using an F-test. Statistically significant differences were determined by Student's *t* test (if data were homoscedastic) or Welch's *t* test (if data were heteroscedastic).

## 3 Results

### 3.1 MTHFD2 expression analysis in various cancers

**Fig. 3:**
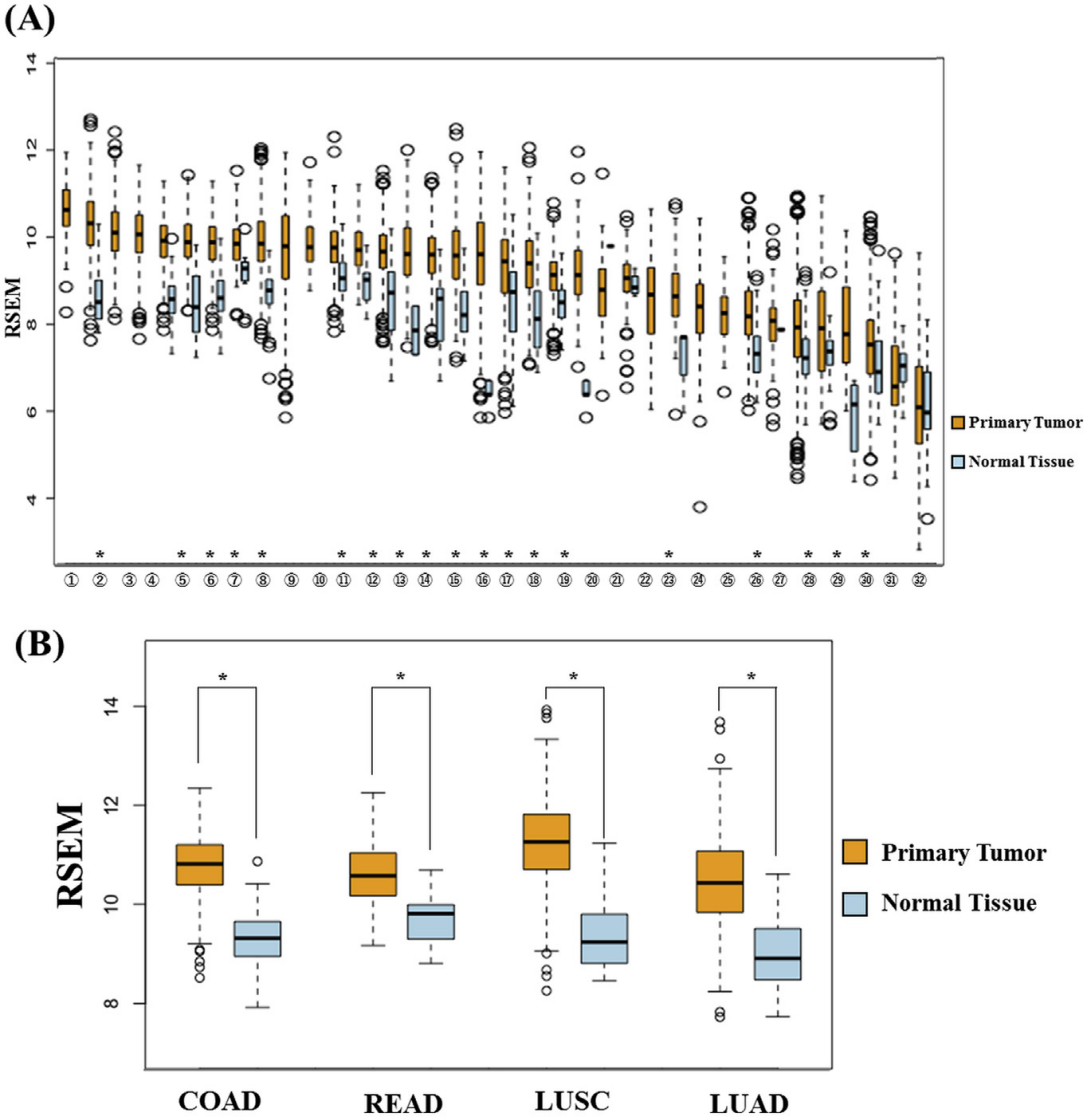
MTHFD2 expression analysis in cancer patients. MTHFD2 expression was examined using clinical data from The Cancer Genome Atlas. (A) Summary of data in various cancers and (B) lung squamous cell carcinoma and colon adenocarcinoma, which would be promising target. The horizontal axis shows cancer types and the vertical axis shows RNA-Seq by Expectation-Maximization (RSEM). The cancer types in the horizontal axis are arranged in order of MTHFD2 expression in primary tumors. (1) Lymphoid neoplasm diffuse large B cell lymphoma (tumor, n = 48; normal, no data); (2) lung squamous cell carcinoma (tumor, n = 501; normal, n = 51); (3) ovarian cancer (tumor, n = 303; normal, no data); (4) testicular germ cell tumors (tumor, n = 150; normal, no data); (5) colon adenocarcinoma (tumor, n = 457; normal, n = 41); (6) cervical squamous cell carcinoma (tumor, n = 304; normal, n = 3); (7) esophageal carcinoma (tumor, n = 184; normal, n = 11); (8) breast invasive carcinoma (tumor, n = 1093; normal, n = 112); (9) low-grade glioma (tumor, n = 516; normal, no data); (10) uterine carcinosarcoma (tumor: n = 57; normal, no data); (11) head and neck squamous cell carcinoma (tumor, n = 520; normal, n = 44); (12) rectum adenocarcinoma (tumor: n = 166, normal, n = 10); (13) sarcoma (tumor, n = 259; normal, n = 2); (14) stomach adenocarcinoma (tumor, n = 415; normal, n = 35); (15) lung adenocarcinoma (tumor, n = 515; normal, n = 59); (16) bladder urothelial carcinoma (tumor, n = 408; normal, n = 19); (17) uterine corpus endometrial carcinoma (tumor, n = 545; normal, n = 35); (18) prostate adenocarcinoma (tumor, n = 497; normal, n = 52); (19) glioblastoma multiforme (tumor, n = 153; normal, n = 5); (20) skin cutaneous melanoma (tumor, n = 103; normal, n = 1); (21) pancreatic adenocarcinoma (tumor, n = 178; normal, n = 4); (22) mesothelioma (tumor, n = 87; normal, no data); (23) pheochromocytoma and paraganglioma (tumor, n = 179; normal, n = 3); (24) adrenocortical carcinoma (tumor, n = 79; normal, no data); (25) uveal melanoma (tumor, n = 80; normal, no data); (26) kidney renal clear cell carcinoma (tumor, n = 533; normal, n = 72); (27) thymoma (tumor, n = 120; normal, n = 2); (28) kidney chromophobe (tumor, n = 66; normal, n = 25); (29) cholangiocarcinoma (tumor, n = 36; normal, n = 9); (30) thyroid carcinoma (tumor, n = 501; normal, n = 59); (31) kidney renal papillary cell carcinoma (tumor, n = 59; normal, n = 29); (32) liver hepatocellular carcinoma (tumor, n = 371; normal, n = 50). *p < 0.01.

MTHFD2 expression was compared between primary tumor and normal tissue in various cancers using TCGA data to determine the specificity of MTHFD2 in cancer cells. The results clarified that MTHFD2 was enhanced in most cancers (Fig. 3). MTHFD2 expression was also enhanced in breast cancer, as previously reported, but was more enhanced in colorectal cancer and lung cancer. These results suggest that MTHFD2 represents a cancer-specific target in these cancers, including breast cancer.

### 3.2 Analysis of the association between cancer prognosis and MTHFD2 expression

**Fig. 4:**
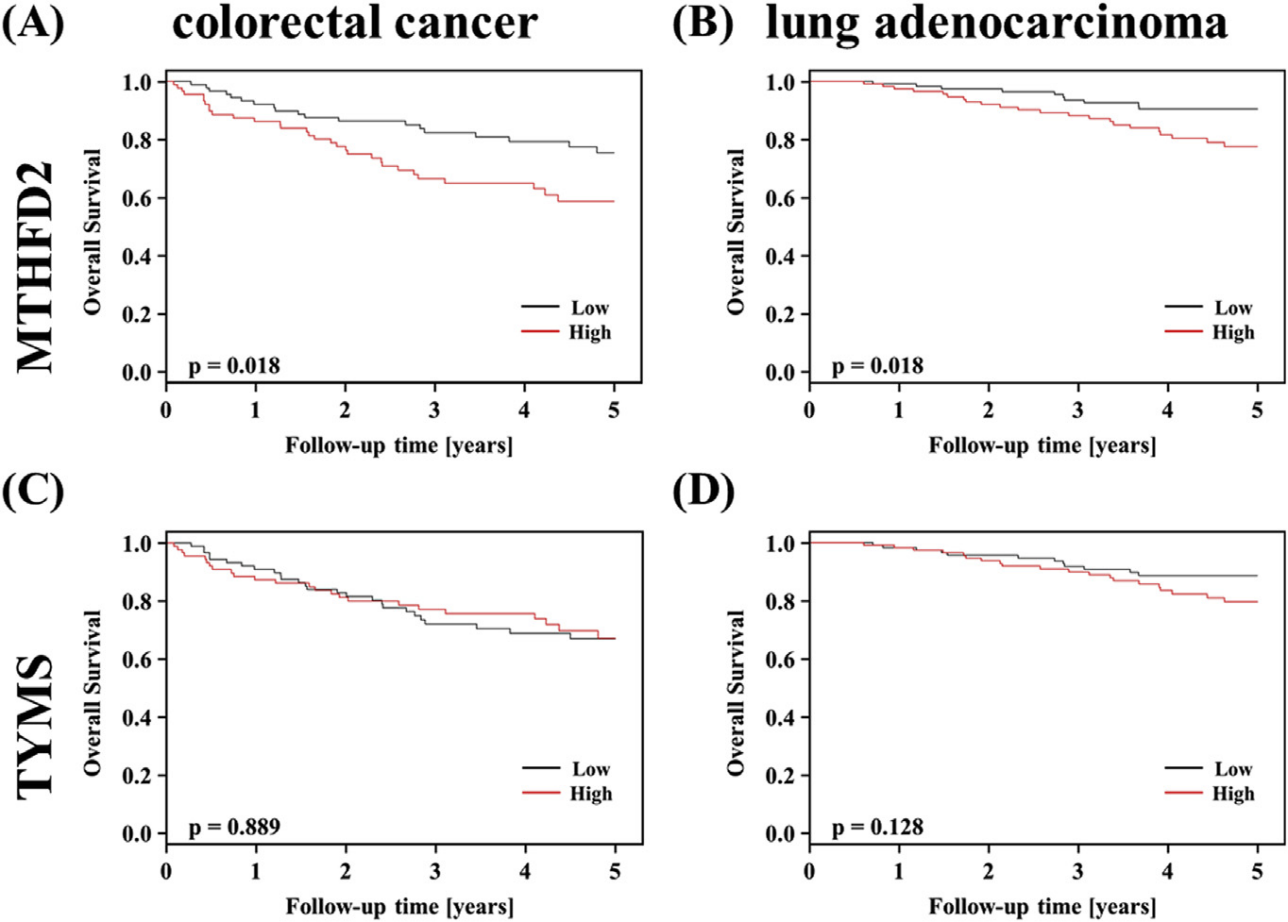
Prognostic analysis. Kaplan–Meier curves of overall survival at 5 years for patients with (**A, C**) colorectal cancer (GSE17536) and (**B, D**) lung adenocarcinoma (GSE31210) according to expression of (**A, B**) MTHFD2 and (**C, D**) TYMS. The horizontal axis shows follow-up time and the vertical axis shows survival rate. Solid line, low-expression group; dashed line, high-expression group.

MTHFD2 expression analysis using TCGA data showed that MTHFD2 expression was enhanced in colorectal and lung cancers. Therefore, we investigated the effect of MTHFD2 expression in each cancer patient using clinical data for colorectal cancer (GSE17536) and lung adenocarcinoma (GSE31210). In both cancers, patients with high MTHFD2 expression had a poor prognosis (Fig. 4A, B). This result suggested that suppression of MTHFD2 is an important target for cancer therapy in colorectal cancer and lung cancer. We also investigated TYMS, an enzyme involved in C1 metabolism and a target of 5-fluorouracil [25]. TYMS expression was not related to overall survival of colorectal cancer or lung cancer patients, suggesting that MTHFD2 has a greater effect on prognosis than TYMS (Fig. 4C, D). These results highlight MTHFD2 inhibitors as a promising therapy for colorectal cancer and lung cancer.

### 3.3 *In silico* screening and identification of important interactions for binding to MTHFD2

**Fig. 5:**
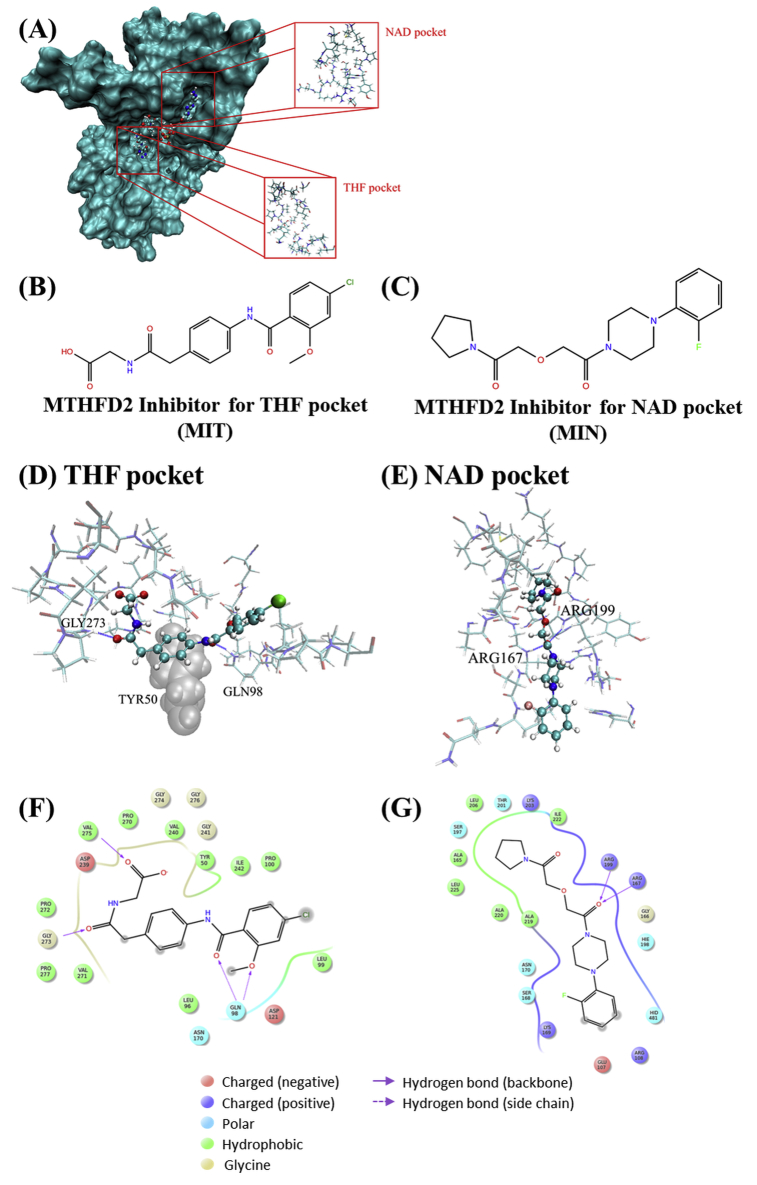
*In silico* simulation for binding to MTHFD2. The structures of the MTHFD2 Inhibitor for (A) THF pocket (MIT) and (B) NAD pocket (MIN). (C) Crystal structure of MTHFD2 (PDB ID: 5TC4). (D) Docking conformation of MIT candidate in the THF pocket. (E) Docking conformation of MIN in the NAD pocket. Each compound is represented by ball-and-stick models with a solid color. The residues around each pocket are represented by sticks with a light color. The residues involved in dispersion force are indicated by van der Waals (balls), shown in gray. The highlighted residues show the important residues for binding to each pocket. The important interactions of (F) MIT and (G) MIN in each pocket. Balls indicate the residues around each pocket.

Since MTHFD2 showed promising results, we screened for MTHFD2 inhibitors using an *in silico* drug discovery technique. MTHFD2 has two active pockets [21]: one is bound by m-THF or f-THF (THF pocket), and the other is bound by NAD(P) or NAD(P)H (NAD pocket). The position of each pocket in the crystal structure of MTHFD2 (PDB ID: 5TC4) is as indicated in Fig. 5A. We performed screening for each pocket using a library of approximately 5 million compounds and detected one candidate compound in each pocket. Each compound was defined as either MIT (MTHFD2 Inhibitor for THF pocket) or MIN (MTHFD2 Inhibitor for NAD pocket) (Fig. 5B, C). The change in free energy (ΔG) caused by MIT binding in the MM/GBSA was greater than that of m-THF, f-THF, and LY345899 (non-specific MTHFD inhibitor) [26] (Table 1). Similarly, the ΔG caused by MIN binding was greater than that of NAD and NADP. Lipinski's rule of five is an index of the physicochemical properties with high oral absorbability. It comprises molecular weight (MW) ≤ 500, log partition coefficient (logP) ≤ 5, hydrogen bond (HB) donor ≤5, and HB acceptor ≤10 [27]. In addition, Rule of Three is also an index of the physicochemical properties that are suitable lead compounds. Rule of Three is MW ≤ 300, logP ≤3, HB donor ≤3, and HB acceptor ≤3 [28]. The physicochemical properties of both candidate compounds mostly matched both indexes, indicating these are promising lead compounds.

**Table 1:** *In silico* score of each metabolite and compound binding to each pocket in MTHFD2.

Target	Metabolites and compounds	ΔG (kJ/mol)	MW	qplogP	HB donor	HB acceptor
THF pocket	m-THF	–85.7	457	0.162	6.25	12.3
	f-THF	–87.3	473	–0.765	7.25	14.3
	LY345899	–74.1	471	0.091	6.25	12.3
	MIT	–169	377	2.54	2.25	7.00
NAD pocket	NAD	–133	665	–2.87	8.00	25.2
	NADP	–112	745	–3.13	9.00	28.5
	MIN	–153	349	0.958	0.00	8.70

G, free energy; MW, molecular weight; HB, hydrogen bond.

The screening for the THF pocket revealed that HBs with Gln98, Gly273, Val275, and van der Waals forces with Tyr50 and Pro277, especially a dispersion force with Tyr50, were identified as important interactions for binding to THF pocket (Fig. 5D, F). Similarly, screening for NAD pocket revealed that HBs with Arg167 and Arg199, and van der Waals forces with Ala165, Leu206, Ala219, Ala220, Leu225 were identified as important interactions for binding to the NAD pocket (Fig. 5F, G). Accordingly, it is important to enhance the interactions with these residues to generate a compound with higher affinity. Using *in silico* screening, the candidate compounds binding to MTHFD2 were detected and the important interactions for binding to MTHFD2 were identified.

### 3.4 The specificity of identified compounds for other enzymes in C1 metabolism

We performed *in silico* docking simulation with MIT and MIN for MTHFD1 and SHMT2 to confirm specificity of MIT and MIN for MTHFD2. As results, ΔG of MIT and MIN for each enzyme were comparable with each metabolite and inhibitor, suggesting MIT and MIN might bind to MTHFD1 and SHMT2 (Table 2). However, ΔG of MIT and MIN for MTHFD2 were greater than that of the metabolite and the inhibitor of MTHFD2. Thus, MIT and MIN would be high specificity for MTHFD2 compared with MTHFD1 and SHMT2.

**Table 2:** *In silico* score of each metabolite and inhibitor for other enzymes.

Protein	Pocket	Metabolites and compounds	ΔG (kJ/mol)
MTHFD1	THF pocket	MIT	–83.5
		m-THF	–75.7
		f-THF	–98.0
		LY345899	–91.5
	NAD pocket	MIN	–101.8
		NAD	–66.8
		NADP	–102.3
SHMT2	-	MIT	–76.0
		MIN	–79.4
		Serine	–54.8
		Glycine	–38.6
		SHIN1	–86.6

### 3.5 The effect on cellular metabolism by treating the candidate compounds

**Fig. 6:**
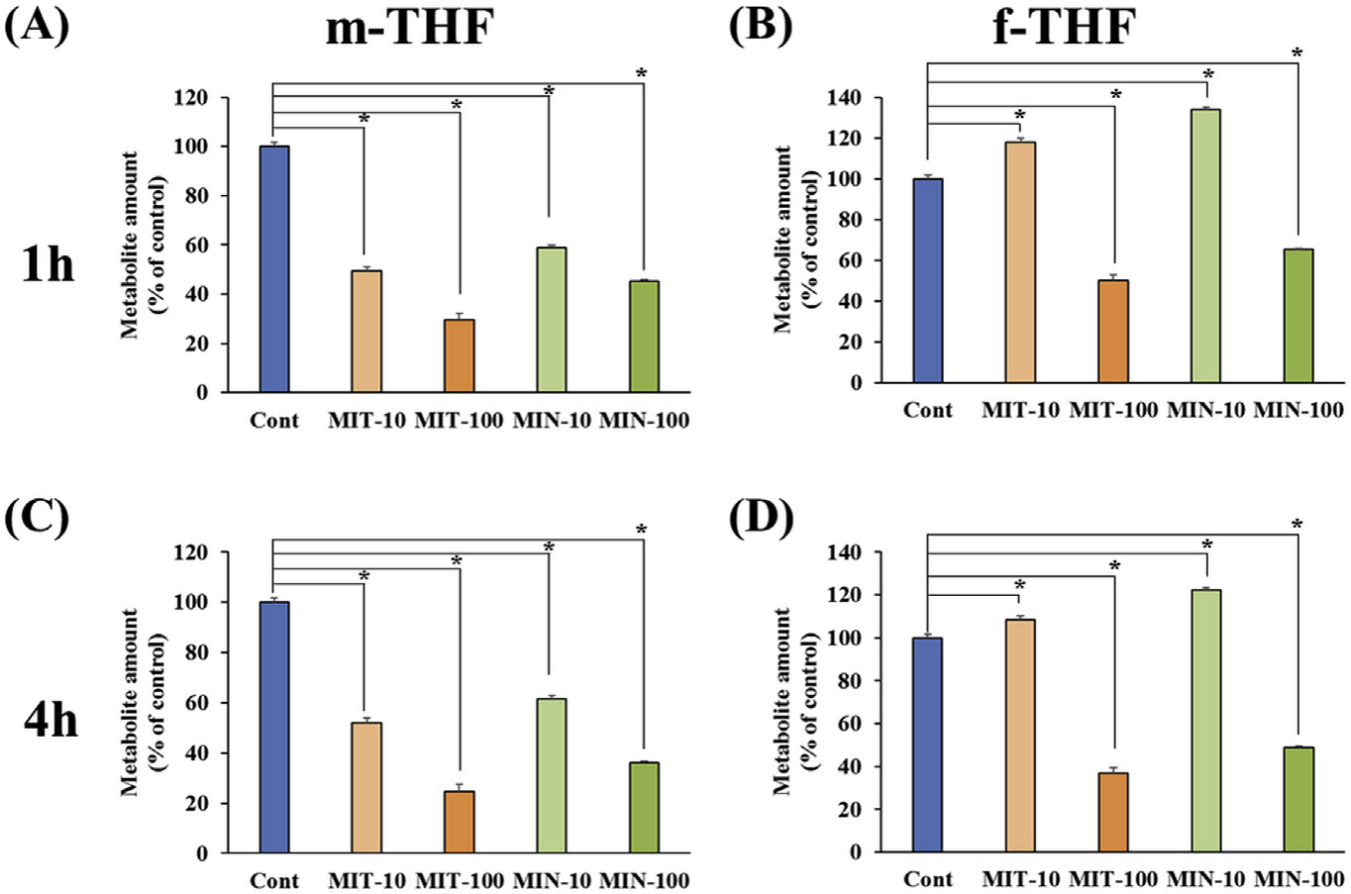
Cell-based assay. Effects of the amount of (A, C) m-THF and (B, D) f-THF in cultured cells following treatment with each candidate compounds, changing the concentration of each compound (10 or 100 μM) at (A, B) 1 h or (C, D) 4 h. The horizontal axis shows the changes in concentration of candidate compounds and treatment time, and the vertical axis shows the ratio of each metabolite compared with untreated cells.

DLD-1 cells were treated with the candidate compounds detected by *in silico* screening. The metabolites were measured to evaluate whether the candidate compounds affected MTHFD2 metabolism. Prior to the cell-based assay, we confirmed that DLD-1 cells highly expressed MTHFD2 using RefExA [29]. MTHFD2 is a bifunctional enzyme that catalyzes the reaction m-THF + NAD(P) ⇌ f-THF + NAD(P)H. Since NAD(P) and NAD(P)H are involved in many pathways, m-THF and f-THF were measured as metabolites. Cells were treated with different concentrations of each candidate compounds (10 or 100 μM) and treatment times (1 or 4 h). Both candidate compounds reduced m-THF regardless of the concentration used (Fig. 6A, C). However, at a concentration of 100 μM, both candidate compounds reduced f-THF, whereas at 10 μM, both candidate compounds elevated f-THF (Fig. 6B, D). These results suggested f-THF may be decreased by partial inhibition of MTHFD2, as there may be compensation from other pathways. Furthermore, the candidate compounds affected the amount of MTHFD2 metabolites.

## 4 Discussion

Metabolism is crucial for cell survival, and differs greatly between cancer cells and normal cells [6]. Anticancer drugs targeted toward cancer cell-specific metabolism minimize the effects on normal cells. Therefore, we focused on MTHFD2, which is highly expressed in cancer cells. In present study, MTHFD2 was enhanced in most cancer types, indicating its potential as a drug discovery target in most cancers. MTHFD2 is highly expressed in colorectal, lung, and breast cancers compared with other cancers. These cancers are reported to have relatively high hypoxia [30]. In hypoxia, oxygen deprivation induces apoptosis by causing a decrease in ATP and an increase in reactive oxygen species [31, 32]. Since MTHFD2 is involved in redox [11], these cancers may adapt to hypoxia by enhancing MTHFD2. In addition, MTHFD2 expression was associated with prognosis of colorectal cancer and lung adenocarcinoma; however, TYMS expression was not. TYMS is an enzyme involved in C1 metabolism and is a target of 5-fluorouracil. It is important for cell survival, including normal cells, but does not affect the malignancy of cancer cells. These results revealed that MTHFD2 is a better indicator of prognosis in cancer patients than TYMS. A previously study showed that expression of DHFR, involved in C1 metabolism and the target of methotrexate, was not associated with prognosis of colorectal cancer and lung adenocarcinoma [16]. Together, these findings emphasize the potential of MTHFD2 as a drug discovery target.

*In silico* screening highlighted MIT and MIN as candidate compounds for the two active pockets of MTHFD2. Both compounds had a higher affinity than the metabolites for each pocket. The two active pockets in MTHFD2 are relatively exposed at the surface, and are assumed to have a lot of contact with the water solvent. Therefore, considering the solvent effect, MM/GBSA seems to reflect the real environment. In addition, both the candidate compounds had physicochemical properties suitable for drug development. This highlights their potential as lead compounds for anticancer drugs targeting MTHFD2. Using *in silico* screening for MTHFD2, the important interactions required to bind to each active pocket (THF and NAD pockets) in MTHFD2 were identified. There are relatively abundant hydrophobic residues in each active pocket, as well as acceptor residues of HBs (Asp121, Asp239, etc.) in THF pocket, and donor residues (Arg167, Lys169, Arg199, Lys293, etc.) of HBs in the NAD pocket. Enhancing interactions with these residues would increase the affinity for compounds.

We evaluated the specificity of MIT and MIN for MTHFD2. As results, MIT and MIN haven't shown high affinity for MTHFD1 and SHMT2. Despite, especially, MIT has the high affinity for MTHFD2, the affinity of that for MTHFD1 was lower than f-THF. The amino acid sequences of MTHFD2 and MTHFD1 are similar, and the residues for binding MIT are conserved. On the other hand, MIT in MTHFD1 didn't interact with several residues including Val275 in MTHFD2. The interaction with these residues might be important to exert the specificity for MTHFD2.

Treatment of cultured cells with each candidate compound resulted in a reduction in MTHFD2 metabolites, depending on the concentration of each candidate compound and the length of treatment. This suggests that C1 metabolism was inhibited, leading to redirection of metabolites to other pathways. At a concentration of 100 μM, both candidate compounds reduced f-THF; however, 10 μM slightly elevated f-THF. At high concentrations of inhibitor, metabolites were diverted to other pathways since the reaction was almost terminated. At low concentrations of inhibitor, however, there may be compensation of metabolites from other pathways. The main metabolites of C1 metabolism include tetrahydrofolate, m-THF, and f-THF. In untreated DLD-1 cells, the amount of tetrahydrofolate, m-THF, and f-THF were 2.06, 13.6, and 0.70 pmol/μg of DNA, respectively. f-THF is very lower amount compared to other metabolites. Moreover, f-THF involved in various reactions such as MTHFD1L, ALDH1L, MTFMT and so on. Thus, it is expected that fTHF easily compensated from other pathway. The consistent results have been obtained for mTHF and fTHF with each MTHFD2 inhibitor, and these results indicate the inhibition of MTHFD2. In the present study, the candidate compounds affected cellular metabolism without optimizing the chemical structural. Modification of the chemical structure of the candidate compounds could produce a more promising MTHFD2 inhibitor.

In conclusion, we validated the possible use of MTHFD2 as a drug discovery target. In addition, we highlighted MIT and MIN as candidate compounds that bind to MTHFD2, and identified the important interactions for binding to MTHFD2. These candidate compounds affected cellular metabolism without improving structural optimization, demonstrating that they could be promising lead compounds targeting MTHFD2. Their activity could be improved based on the important interactions identified in the present study. Our findings may contribute to the development of anticancer drugs targeting MTHFD2, with a view to minimizing the adverse effects caused by anticancer drugs.

## Declarations

### Author contribution statement

Ayumu Asai: Conceived and designed the experiments; Performed the experiments; Analyzed and interpreted the data; Wrote the paper.

Jun Koseki: Conceived and designed the experiments; Analyzed and interpreted the data; Wrote the paper.

Masamitsu Konno: Conceived and designed the experiments; Performed the experiments; Wrote the paper.

Taroh Satoh: Conceived and designed the experiments; Contributed reagents, materials, analysis tools or data.

Tatsunori Nishimura, Noroko Gotoh, Yuichiro Doki, Masaki Mori: Conceived and designed the experiments.

Hideshi Ishii: Conceived and designed the experiments; Wrote the paper.

### Funding statement

This work was supported partially by grants-in-aid for Scientific Research from the JSPS KAKENHI (grant nos. 17H04282, 17K19698, 16K15615, and 15H05791) and the Extramural Collaborative Research Grant of Cancer Research Institute, Kanazawa University (to A.A.).

### Competing interest statement

The authors declare the following conflict of interests: institutional endowments were received by YD, MM, and HI from Taiho Pharmaceutical Co. 20 Ltd., Unitech Co. Ltd. (Chiba, Japan), IDEA Consultants Inc. (Tokyo, Japan), and Kinshu-kai Medical Corporation (Osaka, Japan), and by YD, MM, TS from Chugai Co., Ltd., Yakult Honsha Co. Ltd. and Merck Co. Ltd. Sponsors had no role in the study design or performance, data collection, management, and interpretation, or article preparation and approval.

### Additional information

No additional information is available for this paper.
